# Hepatic rupture from haematomas in patients with pre-eclampsia/eclampsia: a case series

**DOI:** 10.11604/pamj.2018.31.86.15975

**Published:** 2018-10-04

**Authors:** Takura Innocent Kanonge, Felix Chamunyonga, Nellia Zakazaka, Claitos Chidakwa, Mugove Gerald Madziyire

**Affiliations:** 1 Department of Obstetrics and Gynaecology, University of Zimbabwe, College of Health Sciences, Harare, Zimbabwe

**Keywords:** Hepatic haematoma, HELLP syndrome, pre-eclampsia

## Abstract

Hepatic rupture from haematomas is a rare complication of severe preeclampsia/eclampsia especially when complicated with the haemolysis, elevated liver enzymes and low platelet count (HELLP) syndrome. It is associated with poor maternal and foetal outcomes as demonstrated by three cases we describe. The first case had eclampsia at 31 weeks gestation with features of abruptio placentae and at caesarean section we found haemoperitoneum of 1.5 litres, a 10cm liver rupture and a still birth. She subsequently died in ICU within 24 hours of surgery; the second case had eclampsia at 35 weeks and ended up as a table death during emergency caesarean section. She had 4 litres of haemoperitoneum, hepatic rupture, placental abruption and a stillbirth; the third case had pre-eclampsia at 33 weeks with markedly elevated liver enzymes. She had one litre haemoperitoneum, right lobe hepatic rupture and a stillbirth. She recovered after conservative management. Severe pre-eclampsia/eclampsia associated hepatic rupture calls for rapid and aggressive intervention with prompt multidisciplinary management to avert adverse outcomes.

## Introduction

Rupture of a subcapsular hepatic haematoma is a rare complication that occurs in patients with severe preeclampsia or eclampsia especially when complicated with haemolysis, elevated liver enzymes and low platelet syndrome (HELLP). Its incidence ranges between 1:250,000 and 1:40,000 pregnancies but in women with HELLP syndrome its occurrence may be as high as 0.9%-2% [1, 2]. It results from liver engorgement due to extravasation of fluid from porous hepatic microvasculature as a result of abnormal fibrin deposition in the hepatic sinusoids. Sensitization of the reticuloendothelial system of the liver by preeclampsia may render it unable to clear the fibrin thrombi from the circulation resulting in infarction with vascular disruption leading to intrahepatic hemorrhage and parenchymal destruction [3]. Abdominal palpation, manual removal of the placenta, or even uterine contractions have been reported to cause sudden rupture of the subcapsular haematoma.

## Methods

A critical descriptive study of the presentation and management of consecutive patients who presented with hepatic rupture from subcapsular haematoma complicating preeclampsia/eclampsia. This is done through case summaries followed finally with a discussion on the similarities and differences between cases together with how the diagnosis, management and outcomes of these patients compare with global patterns.

## Results

**Case 1**: A 29 year old para 2 gravida 4 at 31+3 weeks gestation was referred from a district hospital with a diagnosis of eclampsia. She was referred with blood pressure (BP) of 179/111mmHg and proteinuria of 2+, and had been commenced on magnesium sulphate and anti-hypertensives. On arrival at the tertiary hospital she had epigastric pain, backache and was drowsy with marked conjunctival pallor. Her BP was 99/77mmHg with a weak thready pulse of 63 beats/minute which was associated with cold peripheries. The uterine fundus was small for gestational age, at 26 weeks size, with marked generalised abdominal tenderness and absent foetal heart tones. She had haemoglobin concentration of 9.3g/dl and thrombocytopaenia of 95*10^9^/L. Induction of labour with amniotomy and oxytocin infusion was commenced. However, the patient immediately deteriorated, with level of consciousness dropping to 3/15 on the Glasgow coma scale, necessitating intubation in the admission area and subsequent emergency caesarean section. Haemoperitoneum of 1500ml was noted with a 10cm laceration on the right lobe of the liver from a subcapsular haematoma which had spontaneously stopped bleeding. The uterus was intact and as anticipated a macerated still born infant was delivered with the placenta showing a retro-placental clot. Massive transfusion of seven units of packed cells and five units fresh frozen plasma was done but the patient deceased within 24 hours of surgery. By the time of her death biochemistry results showed significant hepatic transaminitis and severe thrombocytopaenia of 32*10^9^/L.

**Case 2**: A 26 year old Para 2 Gravida 10 (with 7 first trimester miscarriages) presented at 35 weeks gestational age with a 2 hour history of sudden onset lower abdominal pain associated with vaginal bleeding. She had a caesarean section done in her previous pregnancy at term following a diagnosis of severe gestational hypertension. The current pregnancy had been booked late at 30 weeks and her two antenatal visits had been uneventful except for a single elevated BP reading which had subsequently normalised. On admission she had maternal tachycardia (118 beats/minute) and elevated BP of 140/105 mmHg. She had a woody hard abdomen with generalised tenderness and absence of foetal heart sounds. Blood tests showed a haemoglobin concentration of 13.2g/dl and thrombocytopaenia of 98*10^9^/L. The liver enzymes were normal although renal function showed elevated creatinine (102umol/L) with normal urea. A working diagnosis of pre-eclampsia with severe features was made hence the patient was prepared for an emergency caesarean section but she had an eclamptic convulsion prior to surgery and commencement of magnesium sulphate. She was intubated and taken to theatre but had a cardiac arrest on the table. Perimortem caesarean section done during resuscitation revealed massive haemoperitoneum of 4 litres, 2cm left lobe and 4cm right lobe hepatic ruptures from subcapsular haematomas. A still born infant was delivered showing evidence of retro placental haemorrhage and a couvelaire uterus.

**Case 3**: A 25 year old para 2 gravida 3 was admitted at 33 weeks gestation complaining of epigastric pain, vomiting, lethargy and central chest pain for one day. She had been seen two weeks earlier at a local clinic where a diagnosis of pre-eclampsia with severe features was made following hypertension of 190/100 mmHg and 3+ proteinuria on urine dipstick. An urgent referral to the central hospital was made but the patient was lost to follow up. On admission she had signs of hypovolaemia with pallor, hypotension (88/55mmHg), tachycardia (145 beats per minute) and tachypnoea. Abdominal examination showed epigastric tenderness and a symphysiofundal height of 26 cm. Foetal heart tones were absent on auscultation. Investigations showed haemoglobin concentration of 10.0g/dl, thrombocytopaenia of 96*10^9^/L, urea of 4.1mmol/L and serum creatinine of 118umol/L. Liver enzymes revealed transaminitis with an aspartate aminotransferase (AST) of 1479U/L, alanine aminotransferase (ALT) of 877U/L while urine dipstick showed 4+ of proteinuria. A diagnosis of HELLP syndrome complicated with subcapsular hematoma was made and intraoperative findings were haemoperitoneum (1000ml) arising from hepatic rupture from a subcapsular haematoma in the right lobe of the liver. General surgeons were consulted and they advised conservative management since the haematoma was not enlarging. The uterus was intact prior to the delivery of a macerated stillborn foetus. Postoperatively the patient was managed in intensive care unit with remarkable recovery and resolution of transaminitis and thrombocytopaenia. She required a total of six units of packed cells during her admission and was discharged home on day 11 post caesarean section in good health.

## Discussion

We have presented 3 cases with hepatic rupture from subcapsular haematomas. Two of them had eclampsia associated with maternal deaths while all of them had still births and massive haemorrhage. These findings are consistent with those reported in literature were hepatic rupture from haematomas has been largely reported in patients with preeclampsia and eclampsia leading to severe maternal and neonatal morbidity reported to be 60-86% of cases [4]. HELLP syndrome is a clinical syndrome associated with pre-eclampsia and is characterised by intravascular haemolysis (H), elevated liver enzymes (EL) and low platelet count (LP). The most sensitive marker for haemolysis is reduced serum haptogobulin though peripheral blood smear, serum LDH and elevated indirect bilirubin may all suggest the presence of haemolysis. Some of these tests were not done in our patients partly due to cost and availability but also due to the acuity of the presentation of the three cases. Coagulation tests are important to undertake as they help in ruling our disseminated intravascular coagulation which is an important differential diagnosis of HELLP syndrome. HELLP syndrome commonly occurs at preterm gestations with peak incidence being between 27 and 37 weeks resulting in poor neonatal outcomes due to prematurity and placental abruption [5]. Similarly the 3 cases we described had some features of HELLP syndrome and presented at preterm gestational ages (31-35 weeks). Sibai *et al* reported of high transfusion rates (55%), disseminated intravascular coagulation (21%), acute kidney injury (7.7%) and laparotomy rate of 2% in a prospective study of patients with HELLP syndrome [6]. This is similar to the findings in our series of patients who all required blood products and intensive care admission with two of the patients dying due to significant intra-abdominal haemorrhage. These factors show the need for urgent mobilisation of blood products and intensive care support. According to the American College of Gastroenterology the diagnosis of hepatic rupture from subcapsular haematoma is a clinical one as was demonstrated with regards to our patients. The commonest presentation being in patients with HELLP syndrome who present with acute sudden onset of severe epigastric pain, right upper quadrant pain that radiates to the back associated with signs of hypovolaemia and anaemia. As in our patients presentation is largely antenatal with only a few cases described as occurring in the postpartum period [7].

Imaging has a role where available to confirm the diagnosis through ultrasound scan (USS), computerised tomography (CT) scan or magnetic resonant imaging (MRI). However, due to the rapid deterioration of the clinical condition there is often no time to transfer for imaging as seen in all our patients. This highlights the importance of the use of clinical symptoms and signs in determining the course of management. One of our patients had a cardiac arrest and the other two had hypotension and tachycardia all emanating from acute hypovolaemia. These were indications for urgent resuscitation and surgery which occurred in all three patients albeit with high mortality. Given the high maternal morbidity and mortality associated with hepatic rupture from subcapsular haematoma, prediction tools would be important in guiding timing of intervention. Elevated liver enzymes are the most important pointers of hepatic manifestation. Laboratory parameters of ALT, AST, lactate dehydrogenase (LDH) and serum Uric acid if elevated have been shown to predict a risk of more than 75% serious maternal morbidity in patients with pregnancy induced hypertension [5]. However some authors suggest the clinical features of headache, visual changes, epigastric pain and vomiting have better predictive value for poor maternal outcome [8]. Evidence on the management of patients with hepatic haematoma rupture is largely from a small number of case reports and they all show the importance of multi-disciplinary team care involving obstetricians, paediatricians, intensivists, hepato-biliary surgeons, haematologists and physicians. For haemodynamically stable patients with radiologically confirmed unruptured haematoma as in our third case, conservative non-operative management is appropriate. This requires serial imaging by CT or ultrasound scan together with correction of bleeding disorders often associated with HELLP syndrome through transfusion of blood or its components depending on the clinical scenario [1, 9]. Follow up should be long as spontaneous rupture has been reported to occur six weeks after initial presentation. The clinically unstable patient requires primarily operative management with the options of definitive procedure ranging from peri-hepatic packing and haemostatic angio-embolization to hepatorrhaphy and hepatectomy. A systematic review of case reports conducted by Vigil-De Gracia highlighted a significant improvement in maternal mortality owing to advanced resuscitation, intensive care medicine, surgical intervention, liver transplant and arterial embolization [10].

## Conclusion

The three cases demonstrate the high morbidity and mortality associated with hepatic rupture from subcapsular haematoma, the need for urgent resuscitation, termination of pregnancy, high care and multi- disciplinary care in improving clinical outcomes. This condition is closely associated with pre-eclampsia/eclampsia and HELLP syndrome which all call for urgent haematologic evaluation for end organ dysfunction.

### What is known about this topic

- Hepatic rupture from subcapsular haematoma has high morbidity and mortality;
- Occurrence of hepatic subcapsular haematoma is closely associated with preeclampsia.

### What this study adds

- Early multi-disciplinary care is associated with increased survival chance;
- Care of patients with hepatic subcapsular haematoma is largely governed by clinical features and these should be closely monitored;
- Due to paucity of evidence guiding diagnosis and management, this series adds to the pool of published cases and ultimately may assist in future systematic reviews on this subject.

## Competing interests

The authors declare no competing interests.
